# Tapered arrangement of metallic nanorod chains for magnified plasmonic nanoimaging

**DOI:** 10.1038/s41598-019-39624-1

**Published:** 2019-02-25

**Authors:** Yoshiro Ohashi, Bikas Ranjan, Yuika Saito, Takayuki Umakoshi, Prabhat Verma

**Affiliations:** 1 Department of Applied Physics, Osaka University, Suita, Osaka 565-0871 Japan; 2 Innovative Photon Manipulation Research Team, RIKEN Center for Advanced Photonics, Wako, Saitama 351-0198 Japan; 3 Department of Chemistry, Gakushuin University, Toshima, Tokyo 171-0031 Japan

## Abstract

Plasmonic nanolens, a 3-dimensional tapered arrangement of metallic nanorod chains, holds a great promise as a new plasmonics-based optical nano-imaging technique. While multiple nanorod chains can transfer the near-field signal originating from a sample to an image at a distance larger than a micro-meter, where each nanorod chain contributes in forming one pixel in the image, the tapered arrangement of the nanorod chains with a certain taper angle allows image magnification. We experimentally demonstrate the feature of image formation and magnification in a nanolens by fabricating a tapered arrangement of two silver nanorod chains, which were separated by a distance smaller than the diffraction limit at one end and larger than the diffraction limit at the other end. We placed two nano-sized optical sources of quantum dots near the first ends of the chains, which served as two subwavelength objects. In the optical measurement, we demonstrated that the unresolved subwavelength optical sources could be imaged at the other ends of the chains and were well resolved in accordance with the magnification feature of a nanolens. This verification is an experimental proof of the image magnification, and an important step toward the realization of plasmonic nanolens.

## Introduction

Plasmonics has attracted much attention because it enables one to confine light at the nanoscale through surface plasmon polaritons (SPPs) excited on a metallic nanostructure^1^. Among various applications of such a unique property of plasmonics-based techniques, one of the great applications is super-resolution imaging. Applying this confined light excited at the apex of a metallic nano-tip, super-resolution imaging with the visible light has been demonstrated^2–8^, which was impossible with ordinary far-field optical techniques due to the diffraction limit of light. A recent report theoretically predicted that plasmonic nanolens, which is composed of metallic nanostructures arranged in a certain manner, can be a novel and promising alternative optical imaging technique to achieve the super-resolution^9^.

**Figure 1:**
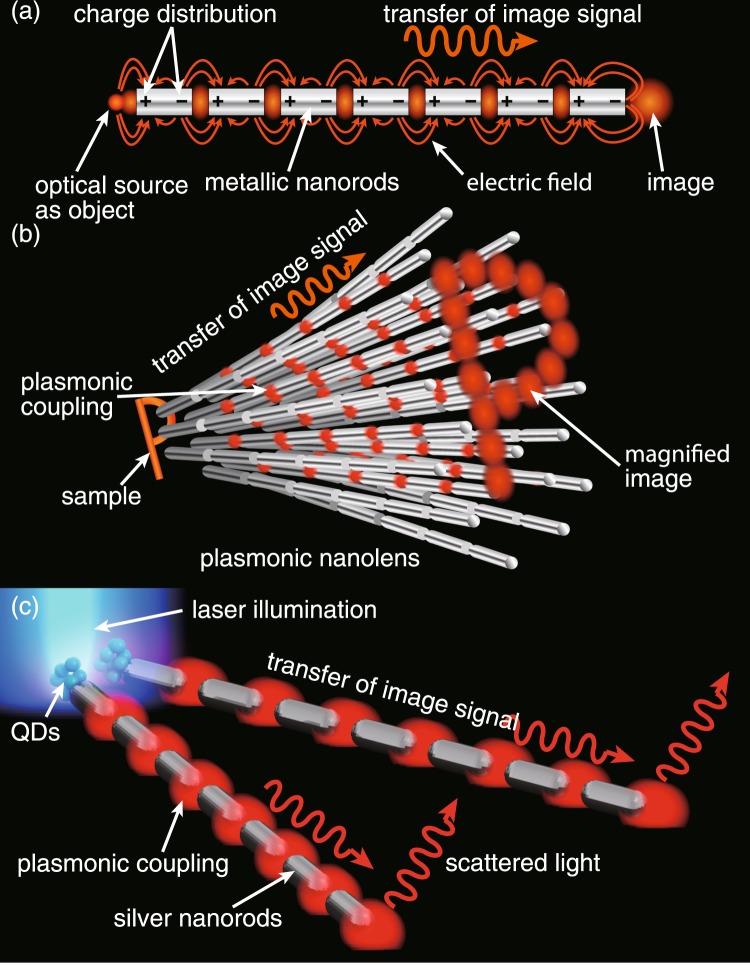
Schematics of the concept of plasmonic nanolens. (**a**) A single metallic nanorod chain that explains the concept of the transfer of image signal through the plasmon coupling between neighboring nanorods. (**b**) A 3D tapered arrangement of metallic nanorod chains, termed as a plasmonic nanolens, which forms a magnified image of a subwavelength sample “P” and works for super-resolution color imaging. (**c**) An illustration of experimental demonstration where two nanorod chains arranged at a certain taper angle form well-resolved images of two QD objects, separated by a distance shorter than the diffraction limit and illuminated by laser light, representing two nano objects separated by a subwavelength distance.

A plasmonic nanolens consists of multiple metallic nanorod chains arranged in a tapered shape. Each nanorod chain in the nanolens can transfer the optical signal originating from a source placed near one end of the chain to the other end via near-field plasmonic interaction^3^ between the neighboring nanorods in the chain. The optical signal originating from the source excites the SPPs in the first nanorod of the chain, which creates near-field light at the other end of the first nanorod. This near-field light excites the SPPs of the second nanorod placed next to the first nanorod in the chain, which creates near-field light at the other end of the second nanorod in a similar fashion. In this way, finally near-field light is created at the end of the last nanorod in the chain, where it can scatter in the far-field. This scattered light, which can be observed in the far-field, contains same optical information as the optical source, and hence represents an image of the source. This process is illustrated in Fig. 1(a). This mechanism explains how a nanorod chain can make an image of a light-emitting source, which is placed near one end of the chain, at its other end. Since this process of image formation relies on the resonant excitation of SPPs within each nanorod, it works only for a source wavelength that matches the SPP resonance, which would depend on the length of individual nanorods as well as on the kind of metal used in the nanorods. Interestingly, as discussed in ref.^9^, a nanorod chain shows an extremely broadened resonance centered at the resonance of an isolated nanorod due to the plasmonic interaction between the nanorods. Therefore, if one carefully selects the metal, the length of individual nanorods and the inter-nanorod gaps, it is possible to have a broad resonance band in a desirable wavelength range. Or, in other words, it is possible to have color imaging through a suitably designed nanorod chain, which would not be possible for a single long nanorod/nanowire^10,11^. The above mentioned mechanism explains the process of image formation of a point source through a single nanorod chain. Multiple nanorod chains can be used to image multiple point sources simultaneously. A sample can be considered as a collection of multiple point sources, where each point source could be imaged in a similar fashion by multiple individual nanorod chains. Each nanorod chain would provide one pixel in the image for one pixel (point source) in the object. If the nanorod chains are arranged in a tapered shape, instead of being parallel to each other, then the pixel-to-pixel separation at the image plane could be larger than that at the object plane. This is analogous to the digital zoom of a camera and hence provides a digitally magnified image of the sample. The magnification in such a system can be defined as the ratio between the pixel-to-pixel separation at the image plane and the pixel-to-pixel separation at the object plane. The magnification will thus depend on the length of the nanorod chain and on the taper angle between the neighboring nanorod chains. If the taper angle is adjusted in a way that at one end, near the sample, the neighboring nanorod chains are separated by a distance smaller than the diffraction limit, while at the other end, the neighboring nanorod chains are separated by a distance much larger than the diffraction limit, then it would be possible to spatially resolve a subwavelength object in its magnified image made by the tapered nanorod chains. The process of magnified imaging via multiple metallic nanorod chains arranged in a 3-dimensional (3D) tapered shape is illustrated in Fig. 1(b), which is called a plasmonic nanolens^9^. The figure depicts a subwavelength-sized sample in the shape of the letter “P” being magnified and imaged through a nanolens formed by a 3D tapered arrangement of multiple nanorod chains. The optical signal picked up by each nanorod chain at the sample end represents individual pixels of the object, which is transferred to the other end of the nanorod chain and works as the corresponding individual pixel in the image. Since the sample is of subwavelength size, it cannot be observed in the far-field. On the other hand, since the pixel-to-pixel separation in the magnified image of letter “P” is larger than the diffraction limit, it can be observed in the far-field where all the image pixels can be spatially resolved and observed by usual far-field optics. This explains how a nano-sized object can be magnified and observed in the far-field through a nanolens made of plasmonic nanorod chains. Image magnification is one of the most important features of a nanolens, because it enables one to “see” a subwavelength object by usual optics. The spatial resolution of a plasmonic nanolens is therefore determined by the separation of nanorod chains at the sample plane. Although shorter separation of nanorod chains at the sample plane would mean higher spatial resolution, the separation smaller than a certain value would cause degradation of the image quality due to a possible crosstalk between neighboring nanorod chains, which may cause mixing up of optical signals from two or more pixels^12^. Recently, super-resolution imaging of subwavelength slits was reported for linear optical system that was illuminated by coherent evanescent light^13,14^. In this case, coherence of optical source affects the resolution in an image, where an analysis on Fourier space provides some analytical understanding on the image formation.

Although the plasmonic nanolens has a great potential as a new super-resolution imaging device, the experimental demonstration and practical applications of a nanolens have still been challenging. This is because the fabrication of a 3D tapered arrangement of nanorod chains in a plasmonic nanolens is very complicated even with the advanced nanolithographic techniques. This is the reason why a plasmonic nanolens has not yet been experimentally demonstrated. In order to show the experimental feasibility of a plasmonic nanolens, we first started with a simplified structure to prove image transfer through a nanorod chain^15^. In our previous study, we demonstrated that a single gold nanorod chain can transfer near-field light originated from a nano-sized optical source of quantum dots (QDs) to a distance much larger than the diffraction limit of light^15^. In that study, we fabricated a nanorod chain by aligning chemically synthesized gold nanorods with template-assisted self-assembly method, and observed an image of QDs placed at one end of the nanorod chain by detecting the scattered light at the other end of the nanorod chain, which was transferred through the plasmonic coupling of the neighboring nanorods in the nanorod chain. This demonstration with 1D structure was the first step to experimentally confirm the concept of a plasmonic nanolens. However, the fabrication technique did not allow us to have a precise control over the uniformity of the length of individual nanorods as well as over the inter-nanorod gaps in the chain. Also, since the plasmon resonance of gold is in the longer wavelength range of the visible spectrum, it is not a perfect choice for full visible range color imaging^16,17^. Nevertheless, we were successful in experimentally demonstrating the first important step to realize a plasmonic nanolens.

Here in this article, we experimentally demonstrated the concept of magnification in a plasmonic nanolens through a 2D tapered arrangement of nanorod chains, utilizing two nanorod chains. At the same time, in order to overcome the issue of precise control in our previous study, we adopted a different and improved technique to fabricate the nanorod chains. First, we chose silver as the nanorod material, which covers broader plasmonic resonance in the visible range than the gold^9,15^. Then, in order to precisely control the length of individual nanorods as well as the inter-nanorod gaps, we synthesized long silver nanowires and milled out some parts of the silver nanowires at equal intervals using focused ion beam (FIB) to fabricate nanorods of desired length with precise gaps between them. The idea of the fabricated tapered arrangement of two silver nanorod chains as well as the transfer of near-field light originated from two closely placed QD sources is illustrated in a schematic in Fig. 1(c). The details of the fabrication process are discussed later. As seen from Fig. 1(c), the two silver nanorod chains are arranged at such a taper angle that they are separated by a distance shorter than the diffraction limit, where two optical sources in the form of QDs are located, while the other ends of the nanorod chains are separated by a distance much larger than the diffraction limit. Through the plasmonic coupling between the neighboring nanorods within each nanorod chains, the light originating from the two QD sources scatters in the far-field at the opposite ends of the nanorod chains. As the origins of the two scattered light spots at the other ends of the nanorod chains are the two QD objects placed near the first ends of the nanorod chains, they carry information of the two QD objects and hence represent the image of the two QD objects. Since the two QD objects are separated by a distance shorter than the diffraction limit, a direct optical observation would not spatially resolve them and thus they will appear as one large object. On the other hand, since their images formed by nanorod chains are separated by a distance larger than the diffraction limit, they can be spatially resolved and can be separately observed by far-field optics.

## Results and Discussions

### Numerical optimization of the fabrication parameters

**Figure 2:**
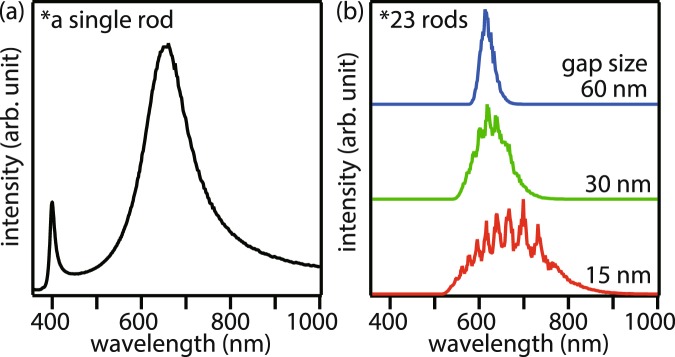
Calculated near-field intensity spectra of (**a**) a single nanorod with 150 nm length and 60 nm diameter and (**b**) a nanorod chain made of 23 similar nanorods with different gap sizes between neighboring nanorods.

In order to correctly fabricate the nanorod chains, we started with a theoretical optimization of the fabrication parameters. For this purpose, we first calculated the near-field intensity spectrum of an isolated single silver nanorod by the finite-difference time-domain (FDTD) method, under the similar conditions that we used in our previous report for a gold nanorod^15^. In the process of optimizing the size of individual nanorods, we found that a 150-nm-long silver nanorod with 60 nm diameter shows plasmon resonance in the center of the visible range as shown in Fig. 2(a). Consequently, we decided to keep this length and diameter of the individual nanorods in further calculation. Since the total length of the nanorod chain and the broadness of the plasmon resonance depend on the number of nanorods, we also optimized the number of nanorods in each nanorod chain for a total length larger than a micrometer and for a considerably broad plasmon resonance to cover a reasonable range in the visible spectrum for a possible color imaging. We concluded that 23 or more nanorods in each chain would give the best results. Further, in order to optimize the inter-nanorod gap size, we calculated the near-field intensity spectra of nanorod chains with different inter-nanorod gaps of 15, 30 and 60 nm, where 23 nanorods with lengths 150 nm and diameters 60 nm were used in the nanorod chain. According to the calculated near-field intensity spectra shown in Fig. 2(b), we found that larger inter-nanorod gap resulted in weaker plasmonic interactions between the neighboring nanorods, resulting in the reduction of broadness in the plasmon resonance, which is not suitable for color imaging. We estimated that the inter-nanorod gap of 15 nm was good for both plasmonic interaction and color imaging. Hence, we concluded that nanorod chains containing 23 individual nanorods of lengths 150 nm and diameters 60 nm with inter-nanorod gaps of 15 nm would make a perfect nanolens for color imaging in the visible range. We therefore decided to adopt the above mentioned dimensions of silver nanorod chains to demonstrate the concept of magnification in the plasmonic nanolens.

### Fabrication of two nanorod chains arranged at a taper angle by FIB milling of long silver nanowires

**Figure 3:**
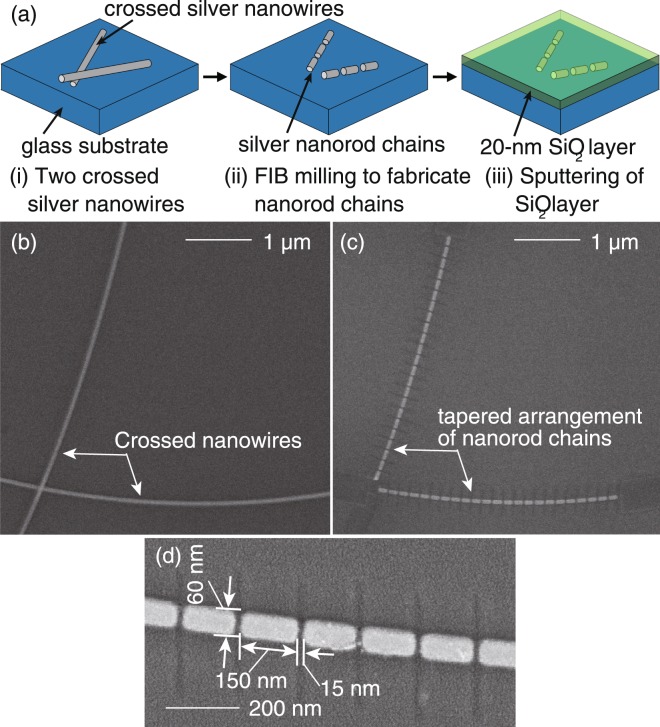
(**a**) (i–iii) Schematics to fabricate a tapered arrangement of silver nanorod chains using FIB. SEM images of (**b**) two crossed silver nanowires and (**c**) a fabricated tapered arrangement of nanorod chains. (**d**) Zoomed SEM image of one of the fabricated nanorod chains.

In order to obtain the nanorod chains, we chemically synthesized a few-micrometer-long silver nanowires with a diameter of 60 nm via one of the polyol processes^18,19^. The details are discussed in Methods. Polyol process is a commonly-used technique to selectively synthesize various shapes of metallic nanoparticles with high quality and high reproducibility using controlled chemical agents^20–22^. Chemical synthesis is advantageous to grow single-crystalline metallic nanostructures with smooth surfaces, which significantly reduces a possible energy loss due to polycrystallinity or surface roughness^23^. After preparing long silver nanowires, two nanorod chains arranged at a taper angle were fabricated by following the process illustrated in Fig. 3(a). By dropping silver nanowires dispersed in ethanol solution on a glass substrate and drying the droplet under the ambient condition, we obtained nanowires distributed on the substrate. We then selected two nanowires that crossed each other from the distributed nanowires as illustrated in Fig. 3(a)(i). At the crossing point, we milled out some parts of the nanowires by FIB in such a way that the remaining nanowires were separated by a distance shorter than the diffraction limit at one end, and at the same time, we cut the nanowires using FIB into nanorods of desired lengths together with controlled inter-nanorod gaps, as illustrated in Fig. 3(a)(ii). Unnecessary parts for the silver nanowires were also removed by FIB to obtain the desired length of the nanorod chain. In this way, a tapered arrangement of silver nanorod chains was obtained. Since silver is degraded by oxidation and sulfurization in ambient atmosphere^24,25^, a 20-nm-thick SiO_2_ layer was subsequently deposited on the fabricated structure with a sputtering system as a protecting layer (Fig. 3(a)(iii)). As examples of structures before and after the fabrication, scanning electron microscopy (SEM) images of the two crossed silver nanowires and the corresponding two nanorod chains fabricated out of them in a tapered shape are shown in Fig. 3(b,c), respectively. Figure 3(d) shows a magnified SEM image of the fabricated silver nanorod chain, where the length and the diameter of individual nanorods were 150 nm and 60 nm, respectively, and the gap between adjacent nanorods was 15 nm, as optimized earlier by simulation.

### Experimental demonstration on the concept of magnification in a plasmonic nanolens

**Figure 4:**
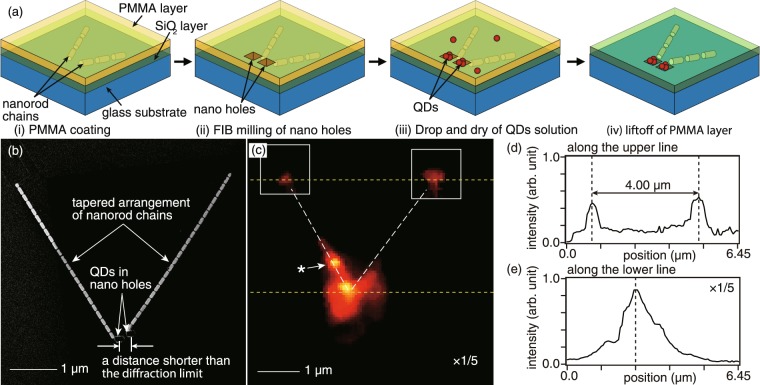
(**a**) Schematics to set QDs as two nano-sized optical sources near one ends of the nanorod chains. (**b**) SEM image of the fabricated tapered arrangement of silver nanorod chains together with the QD samples in two nano-holes near one ends of the chains. (**c**) Scattering image showing an unresolved direct fluorescence from the QDs (bright spot on lower yellow dashed line) and two scattered signals representing the images of the two QD samples (two bright spots on upper yellow dashed line). An additional spot, marked by an asterisk, can also be seen, which corresponds to some unwanted scattering of near-field light. (**d**,**e**) Line profiles along the upper and lower yellow-dashed lines shown in (**c**). The two bright spots in (**d**) are separated by 4.00 µm, which confirms that the image is magnified and well resolved.

Finally, we experimentally demonstrate the concept of magnification in a plasmonic nanolens by resolving two optical sources separated by a distance shorter than the diffraction limit with the help of a fabricated tapered structure of two nanorod chains. We fabricated two nano-sized optical sources by immobilizing core-shell QDs into two nano-holes near one ends of the two nanorod chains via a lift-off process, as illustrated in Fig. 4(a)^26^. Since the two nanorod chains were separated by a distance shorter than the diffraction limit at this end, the two holes were also fabricated within a distance shorter than the diffraction limit. For the immobilization of QDs, we coated a hydrophobic poly-methyl methacrylate (PMMA) layer on the tapered arrangement discussed earlier in Fig. 3, which was coated with a SiO_2_ layer (Fig. 4(a)(i)), and fabricated two nano-holes, each of the size 100 nm square, near the first ends of the two nanorod chains using FIB (Fig. 4(a)(ii)). A small amount of QD solution was then dropped into the two holes and dried under ambient conditions. Since the glass substrate that got exposed within the holes due to the milling process was hydrophilic and the PMMA coating was hydrophobic, the QDs covered with a hydrophilic carboxyl group tend to selectively settle in the holes during the drying process (Fig. 4(a)(iii)). The two QD optical sources were then prepared after lifting off the PMMA layer with acetone, which also removed any possible extra QDs from around the fabricated structure (Fig. 4(a)(iv)). A SEM image of a fabricated tapered arrangement of nanorod chains with two nano-holes holding QDs inside is shown in Fig. 4(b).

In the optical measurement, the QDs were excited by a focused laser beam and the emission from both ends of the nanorod chains was detected using an objective lens and a charge-coupled device (CCD) camera, while the illumination laser was efficiently blocked by using a long-pass filter. An optical image of the fluorescence signal is shown in Fig. 4(c), where one can see four optical spots. The spot on the lower yellow dashed line is direct fluorescence emission of QDs from the two nano-holes. Since the two nano-holes are separated by a distance shorter than the diffraction limit, they appear as one large spot in the optical measurement. The direct fluorescence of QDs is quite strong, thus we decreased the contrast for this spot by five times in the image. The two spots on the upper yellow dashed line are scattered signals from the opposite ends of the two nanorod chains. These two spots, which are shown within two white square, are the images of the two QD sources, which are clearly resolved due to the tapering. It should be noted that, as seen by the SEM image in Fig. 4(b), the two QD sources are clearly separated from each other by a distance larger than 50 nm, as well as from the other nanorod chain by a distance larger than 100 nm, there is no possibility of cross-talk and hence the results show a clear resolution of two unresolved optical sources. Apart from these three spots, one can notice an additional bright spot, marked by an asterisk, which comes from an unwanted scattering spot that may have been created by a dust particle or a protrusion not observed in SEM image. The white-dashed lines indicate the positions of the two nanorod chains. We analyzed line profiles of fluorescent intensity along the two yellow-dashed lines (Fig. 4(d,e)). On the upper yellow-dashed line, the two optical spots are clearly separated by 4.00 µm, as seen in Fig. 4(d), while the two QD sources on the lower yellow-dashed line formed a single unresolved spot of 246 nm in size, as seen in Fig. 4(e). We therefore successfully demonstrated far-field observation of spatially resolved image of two optical sources separated by a diffraction-limited distance through the magnification concept of plasmonic nanolens with the two nanorod chains arranged in a tapered shape.

## Conclusion

As the conclusion, in order to demonstrate the magnification in a plasmonic nanolens, we fabricated a tapered arrangement of two nanorod chains using chemically synthesized silver nanowires and FIB lithography, and immobilized QDs in two nano holes to create two subwavelength optical sources. We then experimentally demonstrated the formation of a magnified image to resolve two optical sources separated by a distance shorter than the diffraction limit by transferring the near-field light to the opposite ends of two silver nanorod chains arranged at a taper angle. The fabricated nanorod chains transferred the near-field light originated from the QDs to a distance longer than micrometer. Apart from the image formation and its transfer to a reasonably long distance, this experimental demonstration of resolving optical sources with the tapered structure is a significant proof of the image magnification by a plasmonic nanolens, which is an important step forward to realize a nanolens. As a prospective goal, if the fabrication techniques in the future allow us to fabricate a 3D tapered structure of nanorod chains, the image magnification of nano-sized samples would become a reality. This would revolutionize super-resolution imaging techniques and would contribute to the diverse nano-science research fields.

## Methods

### Chemical synthesis of long silver nanowires

Long silver nanowires were chemically synthesized to fabricate a tapered arrangement of nanorod chains. Before the synthesis, a flask and a stirring bar were cleaned in aqua regia or HNO_3_ for about 20 min, and rinsed with deionized water for 7-8 times. After dissolving 0.222 g poly vinyl pyrrolidone (PVP) powder with an average molecular weight of 40,000 into 20 mL polyethylene glycol 600 (PEG 600) in the cleaned flask, we added 0.4 mL of 1 M AgNO_3_ aqueous solution in the PVP/PEG mixture. Then, we heated the solution at 95–100 °C in an oil bath for several hours under continuous stirring, which produced silver nanowires in the solution. Finally, after removing the remaining chemicals through the process of centrifuging and washing with acetone and alcohol, silver nanowires dispersed in ethanol solution could be obtained. The existence of oxygen and a precise control of solution temperature played critical roles in selectively synthesizing the desired nanowires. The length of the nanowires could be tuned by the concentrations of the chemicals.

### Fabrication of two silver nanorod chains arranged at a taper angle

In order to obtain two silver nanorod chains arranged at a taper angle, we cut the chemically synthesized silver nanowires by milling with FIB (FB2200, Hitachi High-Technologies) as described in the main text. The fabrication process is shown in Fig. 3(a). After fabricating the tapered structure of two nanorod chains as shown in Fig. 3(c), a 20-nm-thick SiO_2_ layer was subsequently deposited on the fabricated structure with a sputtering system (ACS-4000, ULVAC) as a protecting layer against the degradation of silver by oxidation and sulfurization in ambient atmosphere.

### Immobilization of QDs as optical sources

Two nano-sized optical sources were fabricated by immobilizing core-shell QDs (CdSeTe/ZnS core-shell, Qdot 605 ITK Carboxyl QDs, Invitrogen) into two nano-holes near one ends of the two nanorod chains via a lift-off process as illustrated in Fig. 4(a)^26^. For the immobilization of QDs, we coated a hydrophobic poly-methyl methacrylate (PMMA) (495PMMA A2, Microchem) layer on the tapered arrangement discussed earlier, which was coated with a SiO_2_ layer, and fabricated two nano-holes, each of the size 100 nm square, near the first ends of the two nanorod chains using FIB. 2 µL of an 8-µM QD solution was then dropped into the two holes and dried under ambient conditions. The two QD optical sources were then prepared after drying and lifting off the PMMA layer with acetone, which also removed the possible extra QDs from around the fabricated structure. The final structure together with the QDs is shown in Fig. 4(b).

### Optical measurement

The QDs were excited by a focused laser beam with a wavelength of 473 nm and power of ~100 µW using an oil-immersion objective lens (100×, N.A.: 1.40, Nikon). The emission from both ends of the nanorod chains was collected using the same objective. The illumination laser was efficiently blocked in the measurement by using a long-pass filter (473 nm RazorEdge® Long Pass E-Grade filter, Semrock), and the emitted fluorescence at 602.4 nm was detected using a charge-coupled device (CCD) camera (Pixelfly qe, PCO) for an exposure time of 60 s.
